# Supplementary material for: Choline supplementation: Impact on broiler chicken performance, steatosis, and economic viability from from 1 to 42 days
Source: PLoS One. 2024 Mar 19;19(3):e0295488. doi: 10.1371/journal.pone.0295488 (PMC10950236; doi:10.1371/journal.pone.0295488)
Supplement: Attachment — Submitted filename: PONE-D-23-39073_reviewer.pdf [file pone.0295488.s003.pdf]

PLOS ONE

Choline through diet or supplementation? Synthetic or natural source supplementation? Improving performance, reducing steatosis, and economic viability of broiler chickens from 1 to 42 days.

--Manuscript Draft--

Manuscript Number:	PONE-D-23-39073
Article Type:	Research Article
Full Title:	Choline through diet or supplementation? Synthetic or natural source supplementation? Improving performance, reducing steatosis, and economic viability of broiler chickens from 1 to 42 days.
Short Title:	Choline Supplementation in Broilers: Performance, Steatosis, & Economic Viability (1-42 days)
Corresponding Author:	Matheus Ramalho de Lima<br>Universidade Federal Rural do Semi-Árido<br>Mossoró, RN BRAZIL
Keywords:	hepatoprotective action, natural choline, phosphatidylcholine
Abstract:	This study was carried out to compare the impact of choline supplementation (available from two sources synthetic source and natural sources) on various dosages in broilers. The mode of choline supplementation, via diet and additional sources, synthetic and natural, and the data of performance, carcass quality, blood parameters, and hepatic steatosis were compared. A total of 1050 day-old male Ross 308 broiler chicks were randomly assigned to 10 treatments, with 6 replicates per treatment and 25 birds per replicate. Choline was supplemented using three sources: synthetic choline chloride 60% (CC), and two sources of natural choline A (NCA), and B (NCB). The Control treatment did not receive any choline supplementation. The diets were supplemented with low, intermediate and high doses of choline sources (400g/t, 800g/t, and 1200g/t of CC; 100g/t, 200g/t, and 300g/t of both NCA and NCB). Data analysis was performed using the suitable statistical model to investigate the effects of different doses of choline supplementation (CC, NCA, NCB) on the measured variables. Overall, the results indicated that the the performance of NCA was better than CC & NCB, specifically the dose of 100g/t of NCA outperformed MAR at 100g/t & CC at 400g/t, leading to a significant increase in body weight gain (85.66g & 168.84g respectively), and a noteworthy (9- & 12-point respectively) reduction in feed conversion ratio. Furthermore, NCA contributed to a reduction in steatosis when contrasted with various NCB & CC doses, likely due to the presence of curcumins and catechins in the natural choline source. These findings demonstrated that NCA supplementation yielded superior results compared to CC and NCB across both performance and liver health aspects in broilers aged 1 to 42 days. In conclusion, NCA can be used to replace the CC 60% without compromise on the zootechnical performance in broilers.
Order of Authors:	Matheus Ramalho de Lima
	Isabelle Naemi Kaneko
	Adiel Vieira de Lima
	Lucas Nunes de Melo
	Mario Cesar de Lima
	Anna Neusa Eduarda Ferreira de Brito
	Fernando Guilherme Perazzo Costa
	Andreia D. C. Vilas Boas
	Ana Louise Toledo
	Sigfrido Lopez Ferrer
	Saravanakumar Marimuthu

Powered by Editorial Manager® and ProduXion Manager® from Aries Systems Corporation

Additional Information:	
**Question**	**Response**
**Financial Disclosure**<br><br>Enter a financial disclosure statement that describes the sources of funding for the work included in this submission. Review the submission guidelines for detailed requirements. View published research articles from *PLOS ONE* for specific examples.<br><br>This statement is required for submission and **will appear in the published article** if the submission is accepted. Please make sure it is accurate.<br><br>**Funded studies**<br>Enter a statement with the following details:<br>• Initials of the authors who received each award<br>• Grant numbers awarded to each author<br>• The full name of each funder<br>• URL of each funder website<br>• Did the sponsors or funders play any role in the study design, data collection and analysis, decision to publish, or preparation of the manuscript?<br><br>Did you receive funding for this work?	The author(s) received no specific funding for this work.
**Competing Interests**<br><br>Use the instructions below to enter a competing interest statement for this submission. On behalf of all authors, disclose any competing interests that could be perceived to bias this work—acknowledging all financial support and any other relevant financial or non-financial competing interests.<br><br>This statement is required for submission and **will appear in the published article** if the submission is accepted. Please make sure it is accurate and that any funding sources listed in your Funding Information	The authors have declared that no competing interests exist.

Powered by Editorial Manager® and ProduXion Manager® from Aries Systems Corporation

later in the submission form are also declared in your Financial Disclosure statement.<br><br>View published research articles from *PLOS ONE* for specific examples.<br><br>**NO authors have competing interests**<br><br>Enter: *The authors have declared that no competing interests exist.*<br><br>**Authors with competing interests**<br><br>Enter competing interest details beginning with this statement:<br><br>*I have read the journal's policy and the authors of this manuscript have the following competing interests: [insert competing interests here]*<br><br>* typeset	
**Ethics Statement**<br><br>Enter an ethics statement for this submission. This statement is required if the study involved:<br><br>• Human participants<br>• Human specimens or tissue<br>• Vertebrate animals or cephalopods<br>• Vertebrate embryos or tissues<br>• Field research<br><br>Write "N/A" if the submission does not require an ethics statement.<br><br>General guidance is provided below. Consult the submission guidelines for detailed instructions. **Make sure that all information entered here is included in the Methods section of the manuscript.**	Animal Care and Use Committee (IACUC) from Federal University of Paraiba.

**Format for specific study types**<br><br>**Human Subject Research (involving human participants and/or tissue)**<br>• Give the name of the institutional review board or ethics committee that approved the study<br>• Include the approval number and/or a statement indicating approval of this research<br>• Indicate the form of consent obtained (written/oral) or the reason that consent was not obtained (e.g. the data were analyzed anonymously)<br><br>**Animal Research (involving vertebrate animals, embryos or tissues)**<br>• Provide the name of the Institutional Animal Care and Use Committee (IACUC) or other relevant ethics board that reviewed the study protocol, and indicate whether they approved this research or granted a formal waiver of ethical approval<br>• Include an approval number if one was obtained<br>• If the study involved *non-human primates*, add *additional details* about animal welfare and steps taken to ameliorate suffering<br>• If anesthesia, euthanasia, or any kind of animal sacrifice is part of the study, include briefly which substances and/or methods were applied<br><br>**Field Research**<br><br>Include the following details if this study involves the collection of plant, animal, or other materials from a natural setting:<br>• Field permit number<br>• Name of the institution or relevant body that granted permission	
**Data Availability**<br><br>Authors are required to make all data underlying the findings described fully available, without restriction, and from the time of publication. PLOS allows rare exceptions to address legal and ethical concerns. See the PLOS Data Policy and FAQ for detailed information.	No - some restrictions will apply

A Data Availability Statement describing where the data can be found is required at submission. Your answers to this question constitute the Data Availability Statement and **will be published in the article**, if accepted.<br><br>**Important:** Stating 'data available on request from the author' is not sufficient. If your data are only available upon request, select 'No' for the first question and explain your exceptional situation in the text box.<br><br>Do the authors confirm that all data underlying the findings described in their manuscript are fully available without restriction?	
**Describe where the data may be found in full sentences. If you are copying our sample text, replace any instances of XXX with the appropriate details.**<br><br>• If the data are **held or will be held in a public repository**, include URLs, accession numbers or DOIs. If this information will only be available after acceptance, indicate this by ticking the box below. For example: *All XXX files are available from the XXX database (accession number(s) XXX, XXX.).*<br>• If the data are all contained **within the manuscript and/or Supporting Information files**, enter the following: *All relevant data are within the manuscript and its Supporting Information files.*<br>• If neither of these applies but you are able to provide **details of access elsewhere**, with or without limitations, please do so. For example:<br><br>*Data cannot be shared publicly because of [XXX]. Data are available from the XXX Institutional Data Access / Ethics Committee (contact via XXX) for researchers who meet the criteria for access to confidential data.*<br><br>*The data underlying the results presented in the study are available from (include the name of the third party*	All relevant data are within the manuscript and its Supporting Information files.

and contact information or URL).<br>• This text is appropriate if the data are owned by a third party and authors do not have permission to share the data.<br><br>* typeset	
Additional data availability information:	

Click here to access/download;Manuscript;Submission_PlosOne_2023_11_

Choline through diet or supplementation? Synthetic or natural source supplementation? Improving performance, reducing steatosis, and economic viability of broiler chickens from 1 to 42 days.

Matheus Ramalho de Lima[1¶*], Isabelle Naemi Kaneko[2&], Adiel Vieira de Lima[2&], Lucas Nunes de Melo[2&], Mario Cesar de Lima[2&], Anna Neusa Eduarda Ferreira de Brito[2&], Fernando Guilherme Perazzo Costa[2¶], Andreia D. C. Vilas Boas[3¶], Ana Louise Toledo[3¶], Sigfrido Lopez Ferrer[4¶], Saravanakumar Marimuthu[5¶]

[1] Federal University of Semi-Arid Region, Mossoró, Rio Grande do Norte, Brazil
[2] Federal University of Paraiba, Areia, Paraiba, Brazil
[3] Vidara do Brasil Ltda, Jundiai, São Paulo, Brazil
[4] Vidara Life Ingredients SAU, Barcelona, Espanha
[5] Natural Remedies Private Limited, Bangalore, India

* Corresponding author
E-mail: mrlmatheus@ufersa.edu.br (MRL)

[¶]These authors contributed equally to this work.
[&]These authors also contributed equally to this work.

Abstract

This study was carried out to compare the impact of choline supplementation (available from two sources synthetic ~~source~~ and natural ~~sources~~) on various dosages in broilers. The mode of choline supplementation, *via* diet and additional sources, synthetic and natural, and the data of performance, carcass quality, blood parameters, and hepatic steatosis were compared. A total of 1050 day-old male Ross 308 broiler chicks were randomly assigned to 10 treatments, with 6 replicates per treatment and 25 birds per replicate. Choline was supplemented using three sources: synthetic choline chloride 60% (CC), and two sources of natural choline A (NCA), and B (NCB). The Control treatment did not receive any choline supplementation. The diets were supplemented with low, intermediate and high doses of choline sources (400g/t, 800g/t, and 1200g/t of CC; 100g/t, 200g/t, and 300g/t of both NCA and NCB). Data analysis was performed using the suitable statistical model to investigate the effects of different doses of choline supplementation (CC, NCA, NCB) on the measured variables. Overall, the results indicated that the the performance of NCA was better than CC & NCB, specifically the dose of 100g/t of NCA outperformed MAR at 100g/t & CC at 400g/t, leading to a significant increase in body weight gain (85.66g & 168.84g respectively), and a noteworthy (9- & 12-point respectively) reduction in feed conversion ratio. Furthermore, NCA contributed to a reduction in steatosis when contrasted with various NCB & CC doses, likely due to the presence of curcumins and catechins in the natural choline source. These findings demonstrated that NCA supplementation yielded superior results compared to CC and NCB across both performance and liver health aspects in broilers aged 1 to 42 days. In conclusion, NCA can be used to replace the CC 60% without compromise on the zootechnical performance in broilers.

Introduction

The use of choline in commercial broiler feed is an important aspect in the feed industry to enhances the growth performance and regulates the lipid metabolism as well. Typically, choline plays a significant role in many important metabolic pathways, being crucial for the structural maintenance of cell membranes and organelles, acting as a constituent of phospholipids (Zeisel, 1991; Marimuthu et al. 2019); synthesis of methionine from homocysteine as a labile methyl donor; and a precursor molecule for the formation of acetylcholine in the nervous system. In addition, the choline is a component of very-low-density lipoproteins (VLDL) which involves in triglyceride transport out of the liver and thereby reduces the accumulation of fat in the liver (D'souza et al. 2022; Khose et al. 2019; Selvam et al. 2018; Xu et al. 2010). It also inhibits the fatty acid synthesis by downregulating fatty acid synthase gene expression as well as attenuating its activity (Sherriff JL et al. 2016; Zhu J et al. 2014). Furthermore, choline is referred to as a "lipotropic" factor due to its role in increasing fat utilization, which results in the reduction of fat deposition in the body (Farina et al. 2017; Selvam et al. 2018).

However, the absence of choline, or levels below the recommendation, increases deleterious effects on meat chickens, such as reduction in growth and perosis, especially in younger birds (Selvam et al. 2018; Santiago, et al. 2020). Choline supplementation is commonly done by synthetic sources, but high hygroscopicity and oxidation with loss of vitamins, its corrosive nature, as well as the trimethylamine formation in the intestinal tract of broilers (the common reason less than half of the choline chloride is absorbed), allowed th te scientist to look for an alternative (Zeisel et al. 1986; Calderano et al., 2015).

In this respect, research has been developed to unveil potential substitutes for synthetic sources, and formulations with herbs have been gaining ground with consistent results in hepatoprotective capacity and improvement of performance parameters. These natural products, produced from selected plants and blends of herbs, which can mimic the function of choline (Calderano et al. 2015). Chandrasekaran et. al (2019) when investigating the lipotropic activity of herbal formulations containing *Acacia nilotica* and *Curcuma longa*, demonstrated the lipotropic effect of this natural source which was highlighted by low fat accumulation and anti-lipogenic activity. The formulations of natural choline using herbs such as *Acacia nilotica* and *Curcuma longa* paved a way to get the associating benefits and thus enhancing the effects of hepatoprotective action which was being reported discretely (Yarry et al. 2009, Narayanan et al. 2013). Hence the current study was carried out to compare the impact of choline supplementation (available from two sources synthetic source and natural sources) on various dosages in broilers. The mode of choline supplementation, *via* diet and additional sources, synthetic and natural, and the data of performance, carcass quality, blood parameters, and hepatic steatosis were compared.

Material and Methods

Housing conditions and management

Housing, feeding regimens, and rearing conditions used at the Federal University of Paraiba (UFPB) are thought to be representative of modern commercial broiler operations in Brazil. This trial was carried out at an experimental farm located in Areia – Paraiba, Brazil. Farm facilities comprise 88 identical floor pens. Of these, 42 pens were used. The trial was carried out with 1050 day-old male Cobb 500 broiler chicks, organized in a factorial experiment, 3 x 3 + 1. Chicks were randomly assigned to 1 of 10 treatments, with 6 replicates per treatment and 25 birds per replicate. Individual pens measuring 1.5 x 1.5 m served as experimental units. Pens were consecutively numbered with the respective treatment number using pen cards.

Birds were kept in appropriate (i.e., optimum) environmental conditions (temperature) for age during the trial. Lighting was provided using fluorescent bulbs. The experimental period totaled 42 days. A 3-phase feeding program was used as follows: starter (days 1-21), grower (days 22-35), and finisher (days 36-42). Feed was provided in a pan feeder (capacity, 20 kg). Water was offered *ad libitum* using drinkers.

Standard floor pen management practices were used throughout the experimental period. Birds and facilities were inspected twice daily. The following data were recorded: general health status of broilers, environmental temperature, and constant feed and water supply. Dead birds were removed, and unexpected events were identified. Deaths, including pen number, date of death, bird weight, and potential diagnosis, were recorded using a Daily Mortality Record.

Animals

Day-old male Cobb chicks hatched from eggs produced by 30 weeks-old breeders and weighing 45g on average were obtained from Frango Dourado hatchery (Pernambuco, Brazil). Twenty-five male broiler chicks were housed in each pen. Birds were sexed at the hatchery.

Dietary treatments

Choline was supplemented using three sources: synthetic choline chloride 60% (CC), and two sources of natural choline A (NCA), and B (NCB). The Control treatment did not receive any choline supplementation. The diets were supplemented with low, intermediate and high doses of choline sources (400g/t, 800g/t, and 1200g/t of CC; 100g/t, 200g/t, and 300g/t of both NCA and NCB). Natural Choline A (NCA) is indexed as Kolin Plus (M/s Natural Remedies Pvt Ltd, Bengaluru, India), is a polyherbal formulation containing a combination of *Acacia nilotica* (*A. nilotica*) and *Curcuma longa* (C. longa) belonging to the families of Mimosaceae and Zingiberaceae, respectively. Natural Choline B (NCB) contains mainly *Achyranthes aspera*, *Trachyspermum ammi*, *Azadirachta indica*, and others.

Feed

Mash feeds were manufactured at UFPB Agrarian Center - Campus II, located in Areia, Paraiba, Brazil. Broiler diets were formulated with feedstuffs widely used in Brazil. Diets were representative of local commercial feed formulations and designed to meet or exceeded nutritional requirements for broiler strain and age. Feed batches were independently mixed and bagged. Feed bags were labeled with a trial number, mixing date, type of feed, dietary treatment, and replicate number. Detailed records of feed mixtures and test product inventories were kept. Test feeds were mixed for 4 minutes at 28 rev/min in a 100 kg capacity mixer. The experimental diets were described in Table 1 – 3.

Table 1: Experimental diets from 1 to 21 days

	Investment kg	Basal	CC 400 g/t	CC 800 g/t	CC 1,200 g/t	NCA 100 g/t	NCA 200 g/t	NCA 300 g/t	NCB 100 g/t	NCB 200 g/t	NCB 300 g/t
**Macro Ingredients**											
Corn 7.8%	BRL 1.50	575.1	574.69	574.29	572.89	575	574.9	574.79	575	574.9	574.79
Soybean meal 45%	BRL 3.00	334	334	334	334	334	334	334	334	334	334
Meat meal 38%	BRL 3.00	29	29	29	29	29	29	29	29	29	29
Degummed soybean oil	BRL 6.00	37	37	37	38	37	37	37	37	37	37
Limestone 36%	BRL 0.25	8.2	8.2	8.2	8.2	8.2	8.2	8.2	8.2	8.2	8.2
Common salt	BRL 0.60	4.6	4.6	4.6	4.6	4.6	4.6	4.6	4.6	4.6	4.6
Mineral and vitamin premix 0.4%[1]	BRL 1.00	4	4	4	4	4	4	4	4	4	4
**Micro Ingredients**											
Choline chloride 60%, CC	BRL 13.68		0.4	0.8	1.2						
Natural Choline, NCA	BRL 27.35					0.1	0.2	0.3			
Natural Choline, NCB	BRL 32.82								0.1	0.2	0.3
L-Lysine HCL	BRL 8.00	3.15	3.15	3.15	3.15	3.15	3.15	3.15	3.15	3.15	3.15
DL-Methionine 99%	BRL 18.00	3.7	3.7	3.7	3.7	3.7	3.7	3.7	3.7	3.7	3.7
L-Threonine	BRL 7.00	1.25	1.26	1.26	1.26	1.25	1.25	1.26	1.25	1.25	1.26
**Total Mixed** (kg)		**1,000.00**	**1,000.00**	**1,000.00**	**1,000.00**	**1,000.00**	**1,000.00**	**1,000.00**	**1,000.00**	**1,000,00**	**1,000,00**
**End Price** (BRL/kg)		BRL 2.28	BRL 2.29	BRL 2.29	BRL 2.29	BRL 2.28	BRL 2.28	BRL 2.29	BRL 2.29	BRL 2.29	BRL 2.30
Crude protein	%	21.22	21.22	21.22	21.22	21.22	21.22	21.22	21.22	21.22	21.22
Crude fiber	%	3.35	3.35	3.35	3.35	3.35	3.35	3.35	3.35	3.35	3.35
Calcium	%	0.99	0.99	0.99	0.99	0.99	0.99	0.99	0.99	0.99	0.99
Available phosphorus	%	0.47	0.47	0.47	0.47	0.47	0.47	0.47	0.47	0.47	0.47
Metabolizable energy	kcal/kg	3.140.00	3.140.00	3.140.00	3.140.00	3.140.00	3.140.00	3.140.00	3.140.00	3.140.00	3.140.00
Digestible lysine	%	1.26	1.26	1.26	1.26	1.26	1.26	1.26	1.26	1.26	1.26
Digestible methionine	%	0.65	0.65	0.65	0.65	0.65	0.65	0.65	0.65	0.65	0.65
Digestible methionine + cystine	%	0.93	0.93	0.93	0.93	0.93	0.93	0.93	0.93	0.93	0.93
Digestible threonine	%	0.83	0.83	0.83	0.83	0.83	0.83	0.83	0.83	0.83	0.83
Choline	mg/kg	1.162.65	1.162.49	1.162.54	1.162.60	1.162.60	1.162.54	1.162.49	1.162.60	1.162.54	1.162.49
Phosphatidylcholine	g/kg	-	-	-	-	25	50	75	16	32	48
Sodium	%	0.22	0.22	0.22	0.22	0.22	0.22	0.22	0.22	0.22	0.22
Chlorine	%	0.41	0.41	0.41	0.41	0.41	0.41	0.41	0.41	0.41	0.41

Dollar equivalent to BRL 5.47, July 20, 2022. [1]Minimum premix per kilogram of feed: Mn: 60 g; Fe: 80 g; Zn: 50 g; Cu: 10 g; Co: 2 g; I: 1 g; Se: 250 mg.; Vitamin per kilogram of feed: vitamin A: 15,000,000 IU; vitamin D3: 1,500,000 IU; vitamin E: 15,000 IU; vitamin B1: 2.0 g; vitamin B2: 4.0 g; vitamin B6: 3.0 g; vitamin B12: 0.015 g; nicotinic acid: 25 g; pantothenic acid: 10 g; vitamin K3: 3.0 g; folic acid: 1.0 g.

Table 2: Experimental diets from 22 to 35 days

	Investment kg	Basal	CC 400 g/t	CC 800 g/t	CC 1,200 g/t	NCA 100 g/t	NCA 200 g/t	NCA 300 g/t	NCB 100 g/t	NCB 200 g/t	NCB 300 g/t
**Macro Ingredients**											
Corn 7.8%	BRL 1.50	604.29	603.88	602.48	602.08	604.19	604.09	603.98	604.19	604.09	603.98
Soybean meal 45%	BRL 3.00	299	299	299	299	299	299	299	299	299	299
Meat meal 38%	BRL 3.00	24	24	25	25	24	24	24	24	24	24
Degummed soybean oil	BRL 6.00	49	49	49	49	49	49	49	49	49	49
Limestone 36%	BRL 0.25	8.3	8.3	8.3	8.3	8.3	8.3	8.3	8.3	8.3	8.3
Common salt	BRL 0.60	4.2	4.2	4.2	4.2	4.2	4.2	4.2	4.2	4.2	4.2
Mineral and vitamin premix 0.4%[1]	BRL 1.00	4	4	4	4	4	4	4	4	4	4
**Micro Ingredients**											
Choline chloride 60%, CC	BRL 13.68		0.4	0.8	1.2						
Natural Choline, NCA	BRL 27.35					0.1	0.2	0.3			
Natural Choline, NCB	BRL 32.82								0.1	0.2	0.3
L-Lysine HCL	BRL 8.00	2.93	2.93	2.93	2.93	2.93	2.93	2.93	2.93	2.93	2.93
DL-Methionine 99%	BRL 18.00	3.23	3.24	3.24	3.24	3.23	3.23	3.24	3.23	3.23	3.24
L-Threonine	BRL 7.00	1.05	1.05	1.05	1.05	1.05	1.05	1.05	1.05	1.05	1.05
**Total Mixed** (kg)		**1,000.00**	**1,000.00**	**1,000.00**	**1,000.00**	**1,000.00**	**1,000.00**	**1,000.00**	**1,000.00**	**1,000.00**	**1,000.00**
**End Price** (BRL/kg)		BRL 2.27	BRL 2.28	BRL 2.27	BRL 2.27	BRL 2.27	BRL 2.27	BRL 2.27	BRL 2.27	BRL 2.28	BRL 2.28
Crude protein	%	19.64	19.63	19.63	19.63	19.64	19.63	19.63	19.64	19.63	19.63
Crude fiber	%	3.18	3.17	3.17	3.17	3.18	3.18	3.18	3.18	3.17	3.17
Calcium	%	0.92	0.92	0.92	0.92	0.92	0.92	0.92	0.92	0.92	0.92
Available phosphorus	%	0.43	0.43	0.43	0.43	0.43	0.43	0.43	0.43	0.43	0.43
Metabolizable energy	kcal/kg	3,250.00	3,250.00	3,250.00	3,250.00	3,250.00	3,250.00	3,250.00	3,250.00	3,250.00	3,250.00
Digestible lysine	%	1.15	1.15	1.15	1.15	1.15	1.15	1.15	1.15	1.15	1.15
Digestible methionine	%	0.58	0.58	0.58	0.58	0.58	0.58	0.58	0.58	0.58	0.58
Digestible methionine + cystine	%	0.85	0.85	0.85	0.85	0.85	0.85	0.85	0.85	0.85	0.85
Digestible threonine	%	0.76	0.76	0.76	0.76	0.76	0.76	0.76	0.76	0.76	0.76
Choline	mg/kg	1,083.45	1,262.05	1,440.65	1,619.24	1,083.40	1,083.35	1,083.30	1,083.40	1,083.35	1,083.30
Phosphatidyl choline	g/kg					25	50	75	16	32	48
Sodium	%	0.2	0.2	0.2	0.2	0.2	0.2	0.2	0.2	0.2	0.2
Chlorine	%	0.37	0.37	0.37	0.37	0.37	0.37	0.37	0.37	0.37	0.37

Dollar equivalent to BRL 5.47, July 20, 2022. [1]Minimum premix per kilogram of feed: Mn: 60 g; Fe: 80 g; Zn: 50 g; Cu: 10 g; Co: 2 g; I: 1 g; Se: 250 mg.; Vitamin per kilogram of feed: vitamin A: 15,000,000 IU; vitamin D3: 1,500,000 IU; vitamin E: 15,000 IU; vitamin B1: 2.0 g; vitamin B2: 4.0 g; vitamin B6: 3.0 g; vitamin B12: 0.015 g; nicotinic acid: 25 g; pantothenic acid: 10 g; vitamin K3: 3.0 g; folic acid: 1.0 g.

Table 3: Experimental diets from 36 to 42 days

	Investment kg	Basal	CC 400 g/t	CC 800 g/t	CC 1,200 g/t	NCA 100 g/t	NCA 200 g/t	NCA 300 g/t	NCB 100 g/t	NCB 200 g/t	NCB 300 g/t
**Macro Ingredients**											
Corn 7.8%	BRL 1.50	639.33	637.93	637.53	637.12	638.24	638.14	638.03	638.24	638.14	638.03
Soybean meal 45%	BRL 3.00	264	265	265	265	265	265	265	265	265	265
Meat meal 38%	BRL 3.00	19	19	19	19	19	19	19	19	19	19
Degummed soybean oil	BRL 6.00	55	55	55	55	55	55	55	55	55	55
Limestone 36%	BRL 0.25	8.5	8.5	8.5	8.5	8.5	8.5	8.5	8.5	8.5	8.5
Common salt	BRL 0.60	3.8	3.8	3.8	3.8	3.8	3.8	3.8	3.8	3.8	3.8
Mineral and vitamin premix 0.4%[1]	BRL 1.00	4	4	4	4	4	4	4	4	4	4
**Micro Ingredients**											
Choline chloride 60%, CC	BRL 13.68		0.4	0.8	1.2						
Natural Choline, NCA	BRL 27.35					0.1	0.2	0.3			
Natural Choline, NCB	BRL 32.82								0.1	0.2	0.3
L-Lysine HCL	BRL 8.00	2.75	2.74	2.74	2.74	2.74	2.74	2.74	2.74	2.74	2.74
DL-Methionine 99%	BRL 18.00	2.77	2.78	2.78	2.78	2.77	2.77	2.78	2.77	2.77	2.78
L-Threonine	BRL 7.00	0.85	0.85	0.85	0.86	0.85	0.85	0.85	0.85	0.85	0.85
**Total Mixed** (kg)		**1,000.00**	**1,000.00**	**1,000.00**	**1,000.00**	**1,000.00**	**1,000.00**	**1,000.00**	**1,000.00**	**1,000.00**	**1,000.00**
**End Price** (BRL/kg)		BRL 2.22	BRL 2.23	BRL 2.23	BRL 2.23	BRL 2.23	BRL 2.23	BRL 2.23	BRL 2.23	BRL 2.23	BRL 2.24
Crude protein	%	18.09	18.08	18.08	18.08	18.09	18.09	18.08	18.09	18.09	18.08
Crude fiber	%	3.02	3.02	3.01	3.01	3.02	3.02	3.02	3.02	3.02	3.02
Calcium	%	0.83	0.83	0.83	0.83	0.83	0.83	0.83	0.83	0.83	0.83
Available phosphorus	%	0.39	0.39	0.39	0.39	0.39	0.39	0.39	0.39	0.39	0.39
Metabolizable energy	kcal/kg	3,330.00	3,330.00	3,330.00	3,330.00	3,330.00	3,330.00	3,330.00	3,330.00	3,330.00	3,330.00
Digestible lysine	%	1.04	1.04	1.04	1.04	1.04	1.04	1.04	1.04	1.04	1.04
Digestible methionine	%	0.52	0.52	0.52	0.52	0.52	0.52	0.52	0.52	0.52	0.52
Digestible methionine + cystine	%	0.77	0.77	0.77	0.77	0.77	0.77	0.77	0.77	0.77	0.77
Digestible threonine	%	0.69	0.69	0.69	0.69	0.69	0.69	0.69	0.69	0.69	0.69
Choline	mg/kg	1,007.18	1,185.78	1,364.38	1,542.98	1,007.13	1,007.08	1,007.03	1,007.13	1,007.08	1,007.03
Phosphatidyl choline	g/kg					25	50	75	16	32	48
Sodium	%	0.18	0.18	0.18	0.18	0.18	0.18	0.18	0.18	0.18	0.18
Chlorine	%	0.34	0.34	0.34	0.34	0.34	0.34	0.34	0.34	0.34	0.34

Dollar equivalent to BRL 5.47, July 20, 2022. [1]Minimum premix per kilogram of feed: Mn: 60 g; Fe: 80 g; Zn: 50 g; Cu: 10 g; Co: 2 g; I: 1 g; Se: 250 mg.; Vitamin per kilogram of feed: vitamin A: 15,000,000 IU; vitamin D3: 1,500,000 IU; vitamin E: 15,000 IU; vitamin B1: 2.0 g; vitamin B2: 4.0 g; vitamin B6: 3.0 g; vitamin B12: 0.015 g; nicotinic acid: 25 g; pantothenic acid: 10 g; vitamin K3: 3.0 g; folic acid: 1.0g.

Measurements

Performance

Mortality was recorded daily. Body weight (BW) was recorded on D0, D21, D35, and D42 (end of the experimental period). Feed intake (FI) was recorded on D21, D35, and D42. The feed conversion ratio (FCR; adjusted for mortality) was calculated using body weight gain (BWG) and FI data.

Carcass

On D42, after performance data collection, 5 broilers from each replicate were selected by average weight and euthanized for assessment of carcass characteristics. Abdominal fat, carcass, breast, thigh, and drumstick yield were determined according to BW.

Fatty liver data

On D42, liver samples (1 cm^2) were collected and coated with talc, immediately frozen in liquid nitrogen, and stored in an ultra-freezer. Frozen samples were cut into 20 µm thick sections using a cryostat-microtome for historical slide preparation. Slides were then stained with Sudan IV for lipid quantification. Areas of lipid deposition were stained in red. Samples were scored as follows: 1 - no lipid quantification, 2 - few areas of lipid quantification, 3 - larger areas of lipid quantification, and 4 - lipid quantification over all tissue.

Economic analysis

Combined analysis of liveability (i.e., the final number of birds per treatment), performance, carcass yield, and economic data, which can be adjusted according to natural variations, revealed relative gross margin fluctuations across different scenarios. Using the Control treatment as a reference, the relative gross income derived from breast, thigh, drumstick, and whole chicken sales is 100%. The same applies to the remaining treatments in the same order. Hence, 100 can be used as a benchmark to estimate the difference between treatments.

Liver cholesterol, triglycerides, and lipids

On D42, liver samples (1 cm^2) were collected. One set (Triplicates of ~1 g of tissue) of samples were collected from dispersed zones of the frozen left lobe. The exact wet weight of each sample was determined after thawing and dehydrating the excess moisture on a Whatman filter paper for 10 min at 25°C. Then the total fat was extracted from the liver samples followed by liver triglycerides and cholesterol were quantified using the Folch method (1956), and lipids content using AOAC (2000, Chapter 28.3.09)

Statistical analysis

The statistical analysis of the characteristics studied was performed using the R software, version 4.2.1 (2022). The analysis of variance (ANOVA) assumptions (error normality, random and independent errors, and variance homoscedasticity) was met.

Data analysis was performed using a factorial model to investigate the effects of choline supplementation (CC, NCA, NCB) and doses on the measured variables. A two-way analysis of variance (ANOVA) was conducted to assess the main effects of choline supply and doses, as well as any interactions between them. The significance level was set at α=0.05 for all tests. Post hoc Tukey tests were conducted to compare the means of each choline source and dose to identify specific differences.

Results

There was a significant interaction observed in body weight (BW) (P=0.0405), body weight gain (BWG) (P=0.0394), and feed intake (FI) (P=0.0139), as shown in Table 4. In the overall effect, birds exhibited higher weights with CC supplementation compared to the Without group and NCB (P=0.033), but similar to NCA (P>0.05). Weight gain significantly improved with the intermediate dosage compared to the control (P=0.164), but was similar to the low and high dosages (P>0.05). The BWG data were consistent with the findings regarding final body weight in all aspects. There was no significant effect observed on feed consumption, feed conversion ratio, and survival rate.

Table 4: Effect of choline supplementation on zootechnical parameters of broilers at 21 days of age

**Supplementation**	**IBW**	**BW**	**BWG**	**FI**	**FCR**	**LIVE %**
Without	39.605	866.131b	826.525b	1320.004	1.596	99.123
CC	39.626	894.498a	854.872a	1357.403	1.588	99.415
NCA	39.661	880.030ab	840.370ab	1304.835	1.553	98.538
NCB	39.716	870.052b	830.335b	1336.707	1.610	99.415
**Dosage[1]**	**IBW**	**BW**	**BWG**	**FI**	**FCR**	**LIVE**
Control	39.605	866.131b	826.525b	1320.004	1.596	99.123
Low	39.649	874.488ab	834.839ab	1335.071	1.600	99.123
Intermediate	39.675	892.914a	853.239a	1353.100	1.586	98.538
High	39.678	877.178ab	837.500ab	1310.773	1.565	99.708
**Source**	**0.5521**	**0.0033**	**0.0031**	**0.3046**	**0.3764**	**0.4819**
**Dosage**	**0.88**	**0.0164**	**0.0162**	**0.4921**	**0.7422**	**0.3534**
**Interaction**	**1.000**	**0.0405**	**0.0394**	**0.0139**	**0.1377**	**0.097**
**C.V. (%)**	**0.54**	**2.36**	**2.46**	**6.29**	**6.22**	**1.94**

IBW – Initial body weight; BW – Body weight; BWG – Body weight gain; FI – Feed intake; FCR – Feed conversion ratio; LIVE – Liveability; [1]Dosage means the dose of each choline source, being Low (100g/t for NCA and NCB and 400g/t for CC), Intermediate (200g/t for NCA and NCB and 800g/t for CC), and High (300g/t for NCA and NCB and 1200g/t for CC). a,b Means followed by different letters in the same column differ significantly Tukey test, P<0.05.

Higher body weight (BW) was observed with choline supplementation via CC at the intermediate dosage (P=0.034) and high dosage (P=0.0032), while no significant difference was found at the low dosage (P=0.1232), as evidenced by the data presented in Table 5. Notably, there was no observable effect on the incremental doses with CC supplementation (P=0.0948) or NCA supplementation (P=0.7947). However, NCB supplementation resulted in a significantly higher final weight at the intermediate dosage compared to the low and high dosages (P=0.0150). The BWG data exhibited similar patterns to those observed in BW. Regarding feed intake, significantly higher values were obtained with NCB supplementation compared to NCA (P=0.0161), although no significant difference was observed between NCB and CC (P>0.05). Moreover, NCB showed greater feed intake at the intermediate dosage compared to the high dosage (P=0.0029), while no significant difference was found between the intermediate and low dosages (P>0.05).

Table 5: Effect of interaction between the supplementation sources of broilers at 21 days of age

**BW**	**Control**	**CC**	**NCA**	**NCB**	**P value**
**Control**	866.131				
Low		880.526	882.831	860.106B	0.1232
Intermediate		907.010a	875.366b	896.365abA	0.034
High		895.957a	881.893ab	853.683bB	0.0032
P value		0.0948	0.7947	0.0015	
**BWG**	**Control**	**CC**	**NCA**	**NCB**	**P value**
**Control**	826.525				
Low		840.921	843.182	820.413B	0.1232
Intermediate		867.378a	835.699b	856.637abA	0.0333
High		856.316a	842.226ab	813.955bB	0.003

P value	-	0.094	0.793	0.0015	
**FI**	**Control**	**CC**	**NCA**	**NCB**	**P value**
**Control**	1320.000				
Low		1356.491	1309.424	1339.298AB	0.6188
Intermediate		1357.958ab	1278.326b	1423.014aA	0.0161
High		1357.758	1326.754	1247.807B	0.0739
P value		0.9995	0.601	0.0029	

BW – Body weight; BWG – Body weight gain; FI – Feed intake; a,b Means followed by different letters in the same line differ significantly Tukey test, P<0.05. A, B Means followed by different letters in the same column differ significantly Tukey test, P<0.05.

There was a significant interaction observed in BW (P=0.0164), BWG (P=0.0164), FI (P=0.0339), and FCR (P=0.0097), as presented in Table 6. In the overall effect, feed conversion ratio (FCR) showed higher values with NCB supplementation compared to NCA (P=0.008) but was similar to CC and the Without supplementation (P>0.05).

Table 6: Effect of choline supplementation on zootechnical parameters of broilers at 35 days of age

Supplementation	BW	BWG	FI	FCR	LIVE
Without	2147.639	2108.034	3586.367	1.704ab	96.491
CC	2199.488	2159.862	3648.010	1.690ab	96.491
NCA	2184.211	2144.550	3560.725	1.661b	97.076
NCB	2154.361	2114.645	3671.365	1.736a	97.368
**Dosage[1]**	**BW**	**BWG**	**FI**	**FCR**	**LIVE**
Control	2147.639	2108.034	3586.367	1.704	96.491
Low	2167.512	2127.863	3606.538	1.696	96.491
Intermediate	2188.708	2149.032	3690.273	1.718	96.491
High	2181.841	2142.162	3583.288	1.673	97.953
**Source**	**0.2937**	**0.2929**	**0.1809**	**0.008**	**0.8704**
**Dosage**	**0.6818**	**0.6831**	**0.2054**	**0.2035**	**0.5254**
**Interaction**	**0.0164**	**0.0164**	**0.0339**	**0.0097**	**0.2538**
**C.V. (%)**	**3.67**	**3.74**	**4.44**	**3.73**	**3.56**

IBW – Initial body weight; BW – Body weight; BWG – Body weight gain; FI – Feed intake; FCR – Feed conversion ratio; LIVE – Liveability; [1]Dosage means the dose of each choline source, being Low (100g/t for NCA and NCB and 400g/t for CC), Intermediate (200g/t for NCA and NCB and 800g/t for CC), and High (300g/t for NCA and NCB and 1200g/t for CC. a,b Means followed by different letters in the same column differ significantly Tukey test, P<0.05.

Higher BW was observed with choline supplementation *via* CC at intermediate and high dosages compared to low dosages (P=0.099), as indicated by the data presented in Table 7. The BWG data showed similar effects to those obtained in BW. NCB exhibited higher feed intake at the intermediate dosage compared to the high dosage (P=0.0039) but was similar to the low dosage (P>0.05). The FCR demonstrated better results with NCA compared to CC and NCB at the low dosage (P=0.0425). At the intermediate dosage, the FCR showed higher values with the NCB source compared to the CC and NCA sources (P=0.003). The FCR with the use of CC (P=0.2294) or NCA (P=0.5825) is similar to the evaluated dosages, whereas, with NCB, there is a decrease in effectiveness at the intermediate dosage compared to the other dosages of this additive (P=0.0026).

Table 7: Effect of interaction between the supplementation sources of broilers at 35 days of age

BW	Control	CC	NCA	NCB	P value
**Control**	2147.639				
Low		2115.493B	2216.527	2170.515	0.1008
Intermediate		2251.508A	2153.38	2161.234	0.0708

High		2231.462A	2182.726	2131.332	0.1053
**P value**		0.0099	0.3982	0.6765	
**BWG**	**Control**	**CC**	**NCA**	**NCB**	**P value**
**Control**	2108.034				
Low		2075.888B	2176.878	2130.822	0.101
Intermediate		2211.876A	2113.713	2121.506	0.0706
High		2191.822A	2143.059	2091.604	0.105
**P value**		0.01	0.3981	0.6763	
**FI**	**Control**	**CC**	**NCA**	**NCB**	**P value**
**Control**	3586.367				
Low		3579.563	3577.258	3662.793AB	0.58
Intermediate		3679.007	3552.885	3838.927A	0.0128
High		3685.459	3552.031	3512.373B	0.1592
**P value**		0.4475	0.9536	0.0039	
**FCR**	**Control**	**CC**	**NCA**	**NCB**	**P value**
**Control**	1.704				
Low		1.725a	1.643b	1.719aB	0.0425
Intermediate		1.664b	1.680b	1.810aA	0.0003
High		1.681	1.657	1.679B	0.7663
**P value**		0.2294	0.5825	0.0026	

BW – Body weight; BWG – Body weight gain; FI – Feed intake; FCR – Feed conversion ratio; a,b Means followed by different letters in the same line differ significantly Tukey test, P<0.05. A, B Means followed by different letters in the same collum differ significantly Tukey test, P<0.05.

There was a significant interaction observed in BW (P=0.0166), BWG (P=0.0167), and FCR (P=0.0457), as shown in Table 8. In the overall effect, FCR showed better results with NCA compared to NCB and CC (P=0.0432), and was similar to the Without supplementation (P>0.05).

Table 8: Effect of choline supplementation on zootechnical parameters of broilers at 42 days of age

Supplementation	BW	BWG	FI	FCR	LIVE %
Without	2898.869	2859.263	4756.740	1.665ab	94.737
CC	2938.525	2898.899	4954.271	1.710a	95.029
NCA	2978.035	2938.374	4770.241	1.624b	96.491
NCB	2916.491	2876.775	4884.642	1.699a	96.491
**Dosage[1]**	**BW42**	**BWG42**	**FI42**	**FCR42**	**LIVE42**
Control	2898.869	2859.263	4756.740	1.665	94.737
Low	2950.548	2910.899	4856.164	1.670	95.029
Intermediate	2952.020	2912.345	4912.025	1.686	95.322
High	2930.483	2890.805	4840.964	1.677	97.661
**Supplementation**	**0.3214**	**0.3216**	**0.2392**	**0.0432**	**0.6298**
**Dosage**	**0.7367**	**0.7374**	**0.7143**	**0.9541**	**0.249**
**Interaction**	**0.0166**	**0.0167**	**0.1926**	**0.0457**	**0.8955**
**C.V. (%)**	**3.86**	**3.92**	**6.08**	**5.72**	**4.63**

IBW – Initial body weight; BW – Body weight; BWG – Body weight gain; FI – Feed intake; FCR – Feed conversion ratio; LIVE – Liveability; Means followed by different letters in the same column differ significantly; Tukey test, P<0.05.

Higher BW was observed with choline supplementation *via* NCA at low dosages compared to CC and NCB (P=0.0443), as shown in Table 9. The CC supplementation resulted in higher BW with intermediate and high doses compared to the low dose (P=0.326), while NCA showed better results with the low dose compared to the others (P=0.0203). The NCB was not influenced by the doses (P=0.6306). Low dose weight gain was higher with NCA than with CC (P=0.0445), but similar to NCB. FCR showed better results with NCA compared to NCB (P=0.0405), but similar to CC.

NCA demonstrates similarity to NCB, however, FCR significantly differs between them in the 1-42 day phase (P=0.0405). The similarity of NCA with CC in this variable indicates that they are equivalent in providing choline satisfactorily. However, NCA shows better results with doses of 100g/t to 200g/t, while CC shows better results with doses of 800g/t to 1200g/t.

Table 9: Effect of interaction between the supplementation sources of broilers at 42 days of age

**BW**	**Control**	**CC**	**NCA**	**NCB**	**P value**
**Control**	2898.869				
Low		2866.541bB	3035.380aA	2949.721ab	0.0443
Intermediate		2999.664[a]	2943.436B	2912.958	0.4127
High		2949.368AB	2955.287B	2886.793	0.5159
**P value**		0.0326	0.0203	0.6306	
**BWG**	**Control**	**CC**	**NCA**	**NCB**	**P value**
**Control**	2859.263				
Low		2826.936b	2995.730[a]	2910.028ab	0.0445
Intermediate		2960.033	2903.77	2873.23	0.4122
High		2909.728	2915.62	2847.065	0.5154
P value		0.1329	0.3204	0.6305	
**FCR**	**Control**	**CC**	**NCA**	**NCB**	**P value**
**Control**	1.665				
Low		1.7196	1.6	1.6911	0.0889
Intermediate		1.682ab	1.618b	1.757[a]	0.0405
High		1.729	1.652	1.647	0.2577
**P value**		0.6611	0.6302	0.1446	

BW – Body weight; BWG – Body weight gain; FI – Feed intake; FCR – Feed conversion ratio; a,b Means followed by different letters in the same line differ significantly Tukey test, P<0.05. A, B Means followed by different letters in the same column differ significantly Tukey test, P<0.05.

The breast yield was lower when NCB was used (P=0.0036), as shown in Table 10. On the other hand, this source promoted higher thigh yield (P=0.0251). The leg yield was better with the low dose of choline supplementation (P=0.0065).

Table 10: Effect of choline supplementation on carcass characteristics of broilers at 42 days of age

**Supplementation**	**CY**	**BY**	**LY**	**FY**	**TY**	**DY**
Without	28.472	31.283a	21.247	0.647	9.853b	11.239
CC	28.467	31.388ab	21.383	0.680	9.935ab	11.440
NCA	29.057	31.259ab	21.352	0.680	10.041ab	11.473
NCB	28.936	30.707b	21.548	0.646	10.133a	11.542
**Dosage[1]**						
Control	28.472	31.283	21.247	0.647	9.853b	11.239
Low	29.109	31.213	21.606	0.689	10.150a	11.510
Intermediate	28.676	31.065	21.273	0.665	10.048ab	11.484
High	28.674	31.077	21.405	0.652	9.910ab	11.461
**Supplementation**	**0.0544**	**0.0036**	**0.5018**	**0.623**	**0.0251**	**0.1225**
**Dosage**	**0.1278**	**0.07729**	**0.1832**	**0.6674**	**0.0065**	**0.1938**
**Interaction**	**0.5198**	**0.02**	**0.031**	**0.1141**	**0.5095**	**0.1**
**C.V. (%)**	**5.53**	**4.25**	**5.15**	**32.54**	**5.3**	**5.29**

CY – Carcass yield %; BY – Breast yield %); LY – Leg yield %; FY - Fat yield %; TY – Thigh yield %; DY – Drumstick yield %; Means followed by different letters in the same column differ significantly; Tukey test, P<0.05.

The breast yield was influenced by the supplementation of the high dosage, with a superiority (P=0.0002) of CC and NCA sources compared to NCB, Table 11. Regarding thighdrums yield, there was

a better performance with NCB compared to the others (P=0.0383). NCB performed better with the low dosage than the intermediate (P=0.0017) but was similar to the high in ThighDrums yield.

Table 11: Effect of interaction between the supplementation sources of broilers at 42 days of age

Breast Yield %	Control	CC	NCA	NCB	P value
**Control**	31.283				
**Low**		31.184	31.627	30.827	0.0656
**Intermediate**		31.251	30.962	30.98	0.6401
**High**		31.728a	31.188a	30.313b	0.0002
**P value**		0.2233	0.1429	0.1257	
**Thighdrums Yield %**	**Control**	**CC**	**NCA**	**NCB**	**P value**
**Control**	21.247				
**Low**		21.482ab	21.317b	22.017aA	0.0383
**Intermediate**		21.428	21.394	20.998B	0.249
**High**		21.242	21.343	21.628AB	0.3752
**P value**		0.6804	0.9626	0.0017	

a,b Means followed by different letters in the same line differ significantly Tukey test, P<0.05. A, B Means followed by different letters in the same collum differ significantly Tukey test, P<0.05.

A higher cholesterol content was obtained with the control group compared to the intermediate (P=0.0591), while it was similar in the low and high choline supplementation dosages, as shown in Table 12. Meanwhile, the low dosage promoted a higher liver lipid content compared to the other dosages (P=0.0394). In this context, the supplemented choline sources reduced the steatosis score compared to the diet without choline supplementation (P<0.001), with a reduction observed in all three evaluated dosages compared to the control (diet origin; P<0.001)

Table 12: Effects of treatments on liver parameters of broilers at 42 days of age

Supplementation	LC	LT	LL	SS
Without	187.254	240.118	2.207	3.311a
CC	162.090	305.788	2.719	2.970b
NCA	144.885	215.012	3.151	2.259d
NCB	152.819	238.510	2.659	2.733c
**Dosage[1]**				
Control	187.254a	240.118	2.207b	3.311a
Low	159.284ab	241.614	3.295a	2.867b
Intermediate	131.476b	261.172	2.578ab	2.741b
High	169.034ab	256.524	2.656ab	2.356c
**Supplementation**	**0.3138**	**0.052**	**0.1442**	**<0.001**
**Dosage**	**0.0591**	**0.9167**	**0.0394**	**<0.001**
**Interaction**	**0.6476**	**0.0395**	**0.0623**	**<0.001**
**C.V. (%)**	25.54	31.14	26.63	22.44

LC – Liver cholesterol (mg/dL); LT – Liver triglycerides (mg/dL); LL – Liver lipids (%); SS – Steatosis score; Means followed by different letters in the same column differ significantly; Tukey test, P<0.05.

In the interaction, steatosis showed that it is reduced with the supplementation of an additional source in the diet. However, better results were obtained with NCA and NCB compared to CC (P<0.001), as shown in Table 13. NCA showed a better result than NCB and CC even at the low dosage (P<0.001), consistently achieving better results at the intermediate and high dosages (P<0.001). A lower score was observed at the high dosage of NCA (P<0.001). The liver triglyceride content was higher with CC supplementation compared to NCA (P<0.001), but similar to NCB (P>0.05) at the intermediate dosage.

Table 13: Effect of interaction between supplementation sources of broilers at 42 days of age

Steatosis	Control	CC	NCA	NCB	P value
**Control**	3.311				
**Low**		3.288aA	2.488cB	2.82b	<0.001
**Intermediate**		3.066aA	2.355cA	2.80a	<0.001
**High**		2.555aB	1.933bA	2.577a	<0.001
**P value**		<0.001	<0.001	0.1112	
**Triglycerides**	**Control**	**CC**	**NCA**	**NCB**	**P value**
**Control**	240.118				
**Low**		300.428	242.694	181.718	0.1184
**Intermediate**		361.959a	159.404b	262.154ab	0.004
**High**		254.976	242.938	271.657	0.874
**P value**		0.1708	0.2375	0.2209	

a,b Means followed by different letters in the same line differ significantly Tukey test, P<0.05. A, B Means followed by different letters in the same column differ significantly Tukey test, P<0.05.

Findings from this trial suggest NCA outperformed other sources evaluated. Aside from superior performance, NCA was also able to provide satisfactory results at lower doses. To support use recommendations, economic estimates accounting for the effects of additive and dose on production were presented in Table 14 (Investment per kg of feed per phase and weighted average in days) and Table 15 (Relative Gross Income calculation).

Table 14: Investment per kg of feed per treatment (BRL/kg of feed/treatment) at the different stages of production in broilers aged 1 to 42 days

Age	Control	Choline Chloride 60%			NCA			NCB		
		400 g/t	800 g/t	1,200 g/t	100 g/t	200 g/t	300 g/t	100 g/t	200 g/t	300 g/t
**1-21 d**	2.283	2.288	2.293	2.302	2.286	2.288	2.291	2.286	2.289	2.292
**22-35 d**	2.267	2.272	2.278	2.283	2.270	2.272	2.275	2.270	2.273	2.277
**36-42 d**	2.224	2.231	2.236	2.240	2.228	2.231	2.234	2.229	2.232	2.235
**1 – 42 d**	2.268	2.273	2.278	2.286	2.271	2.273	2.276	2.271	2.274	2.278

Price estimates based on values listed in feed Tables, July 2022.

Combined analysis of liveability (*i.e.*, the final number of birds per treatment), performance, carcass yield, and economic data, which can be adjusted according to natural variations, revealed relative gross margin fluctuations across different scenarios (Table 14).

Using to the Control treatment as a reference, the relative gross income derived from breast, thigh, drumstick, and whole chicken sales is 100%. The same applies to the remaining treatments in the same order. Hence, 100 is a used benchmark to estimate the delta between treatments.

Based on comparative analysis between the control and remaining treatments, NCA fed at 100 g/t yields the highest relative gross income (107.42, *i.e.*, 7.42 p.p higher the than control). Margins derived from NCA at 100 g/ton are also higher relative to the three different doses of Choline Chloride or the NCB product (400 g/t Choline Chloride vs. 100 g/t NCA; 10.54 points; 800 g/t Choline Chloride vs. 200 g/t NCA, 4.06 p.p.; 1,200 g/t Choline Chloride vs. 300 g/t NCA, 4.40 points). NCA fed at 200 g/t, or 300 g/t outperformed the control and Choline Chloride fed at three different doses.

The NCB product was economically superior to the control treatment when fed at 100 g/t and 300 g/t doses, However, 0.6 p.p. losses were estimated at the 200 g/t dose. The NCB product outperformed Choline Chloride fed at 400g/t. In contrast, when fed at 200 g/t, the economic performance of the NCB product was poorer (4 to 5 points less) compared to Choline Chloride fed at 800 g/t or 1,200 g/t (Table 10; highlights in red). Assessment estimates suggest NCA was more efficient than the control treatment, Choline Chloride or NCB at doses used in this trial.

Table 15: Economic assessment

	Control	Choline Chloride, g/t			NCA g/t			NCB, g/t		
		400	800	1,200	100	200	300	100	200	300
**Liveability, %**	94.74	93.86	94.74	96.49	94.74	95.61	99.12	96.49	95.61	97.37
**Weight gain, kg**	2.86	2.83	2.96	2.91	3.00	2.90	2.92	2.91	2.87	2.85
**Feed conversion ratio, kg/kg**	1.67	1.72	1.68	1.73	1.60	1.62	1.65	1.69	1.76	1.65
**Production Factor**	386.21	367.30	397.44	386.40	422.34	408.04	417.02	395.59	371.63	400.03
**Birds Final Adjusted**	142.00	141.00	142.00	145.00	142.00	143.00	149.00	145.00	143.00	146.00
**Feed Intake, kg/treat**	675.46	685.04	707.76	727.93	680.45	672.14	717.96	713.14	722.38	683.89
**Cost of diet, BRL/kg**	BRL 2.27	BRL 2.27	BRL 2.27	BRL 2.28	BRL 2.27	BRL 2.27	BRL 2.27	BRL 2.27	BRL 2.28	BRL 2.28
**Cost of feed, BRL/kg**	BRL 1,531.84	BRL 1,555.31	BRL 1,608.81	BRL 1,657.90	BRL 1,543.97	BRL 1,525.76	BRL 1,630.53	BRL 1,620.70	BRL 1,645.00	BRL 1,560.54
**Broiler production, kg/treat**	411.64	404.18	425.95	427.66	431.02	420.91	440.34	427.71	416.55	421.47
**Cost of Broiler production, BRL/kg**	BRL 3.72	BRL 3.85	BRL 3.78	BRL 3.88	BRL 3.58	BRL 3.62	BRL 3.70	BRL 3.79	BRL 3.95	BRL 3.70
**Price of broiler, BRL/kg**	BRL 7.00	BRL 7.00	BRL 7.00	BRL 7.00	BRL 7.00	BRL 7.00	BRL 7.00	BRL 7.00	BRL 7.00	BRL 7.00
**Breast production, kg/treat**	128.76	126.02	133.11	135.70	136.33	130.31	137.34	131.86	129.05	127.75
**Cost/Breast, BRL/kg**	BRL 11.90	BRL 12.34	BRL 12.09	BRL 12.22	BRL 11.33	BRL 11.71	BRL 11.87	BRL 12.29	BRL 12.75	BRL 12.22
**Price of breast, BRL/kg**	BRL 13.00	BRL 13.00	BRL 13.00	BRL 13.00	BRL 13.00	BRL 13.00	BRL 13.00	BRL 13.00	BRL 13.00	BRL 13.00
**Thigh production, kg/treat**	40.55	40.54	42.38	42.00	43.79	42.81	43.11	43.84	41.78	42.61
**Cost/Thigh, BRL/kg**	BRL 37.78	BRL 38.37	BRL 37.96	BRL 39.48	BRL 35.26	BRL 35.64	BRL 37.82	BRL 36.97	BRL 39.37	BRL 36.62
**Price of the thigh, BRL/kg**	BRL 12.00	BRL 12.00	BRL 12.00	BRL 12.00	BRL 12.00	BRL 12.00	BRL 12.00	BRL 12.00	BRL 12.00	BRL 12.00
**Drumstick, kg/treat**	46.27	46.12	49.03	48.75	49.74	48.19	50.33	49.53	47.86	48.72
**Cost/Drumstick, BRL/kg**	BRL 33.11	BRL 33.73	BRL 32.81	BRL 34.01	BRL 31.04	BRL 31.66	BRL 32.40	BRL 32.72	BRL 34.37	BRL 32.03
**Price of drumstick, BRL/kg**	BRL 12.00	BRL 12.00	BRL 12.00	BRL 12.00	BRL 12.00	BRL 12.00	BRL 12.00	BRL 12.00	BRL 12.00	BRL 12.00
**Gross income, BRL (Breast + Thigh +Drumstick)**	BRL 5,597.15	BRL 5,507.47	BRL 5,809.00	BRL 5,846.65	BRL 5,911.88	BRL 5,732.48	BRL 5,989.08	BRL 5,828.61	BRL 5,669.21	BRL 5,707.02
**Gross margin, BRL (Gross Income - Cost of feed)**	BRL 4,065.30	BRL 3,952.15	BRL 4,200.19	BRL 4,188.74	BRL 4,367.91	BRL 4,206.72	BRL 4,358.55	BRL 4,207.90	BRL 4,024.21	BRL 4,146.48
	100.00	97.17	103.23	102.89	107.42	103.42	107.13	103.53	99.04	102.07
		100.00	106.23	105.89	110.54	106.43	110.24	106.55	101.92	105.04
			100.00	99.68	104.06	100.19	103.78	100.30	95.95	98.88
				100.00	104.40	100.52	104.11	100.62	96.25	99.20
**Relative Gross margin, %**					100.00	96.28	103.58	96.64	95.66	103.06
						100.00	103.58	100.10	95.76	98.69
							100.00	96.64	92.45	95.28
								100.00	95.66	98.59
									100.00	103.06

Color scale: color changes indicate a 10% change in value. Red: lowest value. Dark green: highest value. Yellow, orange, light green: 10% value difference intervals. Benchmark: 100.

Discussion

During the total period of this trial (1-42 days), statistical differences were observed for the performance variables evaluated by Tukey analysis. Furthermore, employing orthogonal contrasts, it was evident that NCA exhibited superior performance compared to the NCB product. Specifically, NCA demonstrated notable enhancements in terms of body weight (BW) with an increment of 61.54 g/bird ($P = 0.0143$), body weight gain (BWG) with an increment of 61.59 g/bird ($P = 0.0142$), and feed conversion ratio (FCR) with an improvement of 7.6 points ($P = 0.0190$). Furthermore, birds fed with NCA displayed a trend of lower feed intake by 114.4 g/bird, although this difference was not statistically significant. This aligns with a study by Khose et al. (2018) that suggested herbal choline supplementation at 0.350 and 0.500 kg/ton of feed can effectively substitute synthetic choline chloride-60%, typically used at 1 kg/ton of broiler feed. Similarly, Calderano et al. (2015) evaluated the use of a vegetal source of choline in five levels of supplementation as a replacement of choline chloride at 60% and did not find any difference between the sources on the performance parameters, but they concluded that the use of 100 mg.kg-1 of a vegetal source of choline to replace the use of choline chloride in corn-soybean meal diets for broilers from 1 to 42 days of age. Likewise, Kathirvelan et al. (2013) reported comparable body weight outcomes for both natural and synthetic choline-supplemented groups. Correspondingly, Selvam et al. (2018) demonstrated that the NCA at 400 g/ton replaced the function of 1 kg/ton of synthetic choline (choline chloride 60%) in broilers, showing the potential to mimic biological activities of synthetic choline through the restoration of negative effects caused by a choline-deficient diet. On the other hand, Zhang et al. (2021) investigated the effects of replacing synthetic choline with a natural choline source and demonstrated no significative difference on zootechnical performance and nutrition utilization and carcass characteristics. However, the current results clearly demonstrates the importance of choline supplementation and additionally, the results demonstrate the superiority of natural sources over synthetic ones, with significant results, especially with NCA.

Exploring carcass characteristics, the study revealed that at 200g/t, NCA led to an increase in leg weight, while at 300g/t, it caused a decrease in thigh weight and an increase in breast weight compared to NCB at 300g/ton. Additionally, NCA at 200g/ton exhibited a higher carcass yield compared to Choline chloride at 800g/t. These findings were corroborated by Khose et al. (2019), which demonstrated the advantages of herbal choline supplementation at 0.5 kg/ton of feed in terms of enhanced liver protection, carcass traits, and economic viability in broiler production. Moreover, a comparative analysis was conducted among NCA, choline chloride, and NCB. Significant distinctions emerged between NCA versus Choline chloride and NCA versus NCB in terms of controlling fatty liver. NCA exhibited greater efficacy in mitigating fatty liver incidence in broilers compared to choline chloride and NCB, showing a dose-dependent effect. In this study, NCA at 200 g/t or 300 g/t outperformed the Control and Choline Chloride at various doses. In a similar vein, Gangane et al. (2010) reported reduced fatty liver incidence in broilers through herbal choline feeding. NCA contains certain compounds with proven hepatoprotective effects, such as polyphenols & curcuminoids. Therefore, findings from this trial, particularly the significant reduction in fatty liver development, may be attributed to curcumin and catechin contained in the natural source of choline employed. Studies have shown that curcumin supplementation can improve the growth performance of broiler chickens (Zhang et al., 2017; Upadhaya et al., 2016; Huang et al., 2015; Zhang et al., 2018). Moreover, the phytoconstituents (polyphenols and curcuminoids) present in NCA modulates the liver genes accounts for fat metabolism/catabolism and lipogenesis (Marimuthu et al., 2022). This might enhance the energy availability at the muscular level which inturn enhanced fat utilization. Because of that, the NCA outperformed CC and NCB, as shown by better animal performance parameters and best carcass yields.

As anticipated before, combined analysis of performance data and economic estimates suggest a beneficial effect of NCA compared to remaining treatments, and that NCA given at the 100 g/t dose yields the highest relative gross margin. Economic assessment findings estimate to support the superiority of NCA relative to NCB and CC in this trial in terms of animal performance as well as carcass and cut yield in broilers aged 1 to 42 days, particularly when fed at 100 g/t. In this study's conditions, the dosage of 100g/ton probably was enough to attend to the broilers' requirements due to the low oxidative stress conditions and the use of a corn-soy-based diet. In field conditions with challenges such as low quality of ingredients, heat stress, and high density of animals, higher doses of the NCA evaluated in this study (200g/t or 300g/t) can be recommended. From a practical standpoint (i.e., product storage and feed logistics) the lower dose requirement is another advantage of NCA relative to Choline Chloride, the commercial doses recommended of CC (400, 800, or 1,200 g/t) can involve several space-related and logistics concerns. Therefore, the incorporation of NCA at a dosage of 100g/t in broiler feed could serve as a viable substitute for choline chloride and NCB.

Conclusion

Natural Choline A is a more effective source of supplemental choline relative to the other sources evaluated. Natural Choline A is a safe source of choline with enhanced hepatoprotective effects, which ensures better animal performance and carcass quality. Natural Choline A also has greater economic efficiency compared to Chloride Choline and Natural Choline B. The dose of 100 g/t of Natural Choline A is recommended in low oxidative stress conditions. In higher exposure to heat stress, it is recommended doses of 200g/ton or 300g/t of Natural Choline A in the diet to broilers from 1 to 42 days of age.

References

1. AOAC International. (2000). Official methods of analysis of AOAC International (Vol. 1). AOAC International. Chapter 28.3.09: Fat (Crude) or Ether Extract in Animal Feed.
2. CALDERANO, A.A. et al. RAMALHO, R.V.N.; RODRIGUES, J.B.; CESAR, R.A. Cienc. anim. bras. v.16, n.1, p. 37-44, jan./mar. 2015 https://doi.org/10.1590/1089-6891v16i127404
3. CHANDRASEKARAN, P. R.; MURUGAN, S.; RICHARD, E.J.; BETHAPUDI, B.; PURUSOTHAMAN, D.; VELUSAMI, C.C.; D’SOUZA,P.; MUNDKINAJEDDU, D.; TALKAS, M. S. Evaluation of Lipotropic Effect of Herbal Formulation on Hepatic Fat Accumulation in Rats Fed with Methionine-Choline Deficient Diet. Pharmacognosy Magazine, Volume 15, Issue 66, October-December 2019 (Supplement 3)
4. D’SOUZA, P.; SELVAM, R. Evaluation of polyherbal formulation in broilers fed high energy diet: Implications on zootechnical parameters, fat accretion, and serum L-carnitine levels *D’souza and Selvam / J. Adv. Vet. Anim. Res., 9(1): 166–174, March 2022*
5. Farina, G., Kessler, A. D. M., Ebling, P. D., Marx, F. R., César, R., & Ribeiro, A. M. L. (2017). Performance of broilers fed different dietary choline sources and levels. *Ciência Animal Brasileira*, *18*, e37633.
6. FOLCH, J.; LEES, M.; STANLEY, G.H.S. A simple method for the isolation and purification of total lipids from animal tissues. J Biol Chem. (1957) 226:497–509. doi: 10.1007/s10858-011-9570-9.

7. Gangane, G. R., Gaikwad, N. Z., Ravikanth, K., & Maini, S. (2010). The Comparative effects of synthetic choline and herbal choline on hepatic lipid metabolism in broilers. *Veterinary World*, *3*(7), 318.
8. HUANG, J., ZHONG, X., WANG, Y., & ZHANG, J. (2015). Effects of dietary curcumin supplementation on growth performance, intestinal morphology, and nutrient utilization of broiler chickens. Journal of animal science and biotechnology, 6(1), 1-7.
9. Kathirvelan, C., Chandrasekaran, D., Vasanthakumar, P., & Purushothaman, M. R. (2013). Effect of replacement of synthetic choline with herbal choline on growth performance of broilers. *Indian Journal of Animal Nutrition*, *30*(2), 184-187.
10. Khose, K. K., Manwar, S. J., Gole, M. A., Ingole, R. S., & Rathod, P. R. (2018). Efficacy of herbal choline as a replacement of synthetic choline chloride in diets on growth performance of broilers. *J Lives Res*, *8*(10), 313-322.
11. KHOSE, K.; MANWAR, S.; GOLE, M.; INGOLE, R.; AND RATHOD, P. 2019. Replacement of Synthetic Choline Chloride by Herbal Choline in Diets on Liver Function Enzymes, Carcass Traits and Economics of Broilers. Journal of Animal Research: v.9 n.1, p. 87-93.
12. MARIMUTHU, S. and D'SOUZA, P. Field performance of Kolin Plus, a polyherbal formulation to replace synthetic choline chloride. International Journal of Veterinary Sciences and Animal Husbandry 2019; 4(5): 32-34
13. NARAYANAN K, KUNNATHUR MS, CHANDRASEKARAN G. Protective effect of Acacia nilotica (L.) against acetaminophen-induced hepatocellular damage in Wistar rats. Adv Pharmacol Sci. 2013;2013.
14. SANTIAGO, G.S.; VIEIRA, S.I.; STEFANELLO, C.; SIMOES, C.T.; KINDEIN, I.; MARIA, D.D.; IBAIRRO, P. Dietary choline affects field performance and broiler leg deviations. Livestock Science 240 (2020) 104127
15. SELVAM R, SARAVANAKUMAR M, SURESH S, CHANDRASEKERAN CV, PRASHANTH D. Evaluation of polyherbal formulation and synthetic choline chloride on choline deficiency model in broilers: implications on zootechnical parameters, serum biochemistry, and liver histopathology. Asian-Australas J Anim Sci. 2018 Nov;31(11):1795-1806.
16. Marimuthu, S., Suresh, S., & D'Souza, P. (2022). Identification of lipid regulatory genes modulated by polyherbal formulation in chicken liver tissues using transcriptome analysis. *Journal of advanced veterinary and animal research*, *9*(3), 432.
17. SHERRIFF, J.L., O'SULLIVAN, T.A., PROPERZI, C., ODDO, J.L., ADAMS, L.A. Choline, its potential role in nonalcoholic fatty liver disease, and the case for human and bacterial genes. Adv Nutr 2016;7:5‑ 13.
18. UPADHAYA, S. D., PARK, J. W., & LEE, J. H. (2016). Effects of curcuminoid supplementation on growth performance, nutrient digestibility, meat quality, the relative weight of organs and economics in broiler chickens. Italian Journal of Animal Science, 15(2), 313-318.
19. XU, C.F., YU, C.H., XU, L., SA, X.Y. AND LI, Y.M. 2010. Hypouricemic therapy: a novel potential therapeutic option for nonalcoholic fatty liver disease. Hepatology, in the press, doi: 10.1002/hep.23798.
20. YARRU LP, SETTIVARI RS, GOWDA NKS. Effects of turmeric (Curcuma longa) on the expression of hepatic genes associated with biotransformation, antioxidant, and immune systems in broiler chicks fed aflatoxin. Poult Sci. 2009; 88:2620–7.
21. ZEISEL SH, DACOSTA KA. Increase in human exposure to methylamine precursors of N-nitrosamines after eating fish. Cancer Res. 1986; 46:6136–8.
22. , J.K. Formation of methylamines from ingested choline and lecithin. Journal of

Pharmacology and Experimental Therapeutics. 1983;225:320-324.Disponível em: http://jpet.aspetjournals.org/content/225/2/320.long. ACesso em 25 janeiro 2015.

23. ZHANG, L., XU, Y., ZHAO, L., & CHEN, X. (2017). Effects of dietary curcumin supplementation on growth performance, intestinal morphology, and nutrient utilization of broiler chickens. Journal of applied poultry research, 26(2), 175-182.

24. ZHANG, X., YANG, Z., XIE, J., JI, J., & LIU, L. (2018). Effects of curcumin supplementation on antioxidant status, inflammatory cytokines, and heat shock protein 70 in broiler chickens subjected to heat stress. Poultry Science, 97(8), 2730-2736.

25. ZHANG, Y., WANG, J., LI, Y., & CHEN, J. (2015). Effects of dietary phosphatidylcholine supplementation on growth performance, carcass characteristics, meat quality, and lipid metabolism in broiler chickens. Journal of the Science of Food and Agriculture, 95(9), 1820-1826.

26. ZHU J., WU, Y., TANG .Q, LENG Y., CAI W. The effects of choline on hepatic lipid metabolism, mitochondrial function, and antioxidative status in human hepatic C3A cells exposed to excessive energy substrates. Nutrients 2014;6:2552‑ 71.

Dear Reviewers,

Thank you for your thorough evaluation of our manuscript titled "Choline through Diet or Supplementation? Synthetic or Natural Source Supplementation? Improving Performance, Reducing Steatosis, and Economic Viability of Broiler Chickens from 1 to 42 Days." We appreciate your valuable feedback and have made the necessary revisions based on your comments.

We apologize for any oversight regarding the similarity to the previously submitted manuscript (PONE-D-23-09736). We acknowledge that this is indeed a resubmission.

Formatted: No bullets or numbering

We hope that these changes address the concerns raised during the initial review. Please find the revised manuscript attached. We appreciate your time and effort in evaluating our work.

Significant adjustments have been made to the document, and the addressed items were met punctually. Additionally, points that were not previously included in the discussion have been added. Therefore, some aspects do not have a direct response to the specific item, as the context of our revision has evolved. We conducted a broader edit, considering the points raised, and in some cases, even altering the structure from the previously submitted version.

If you have any further suggestions or require additional clarifications, please do not hesitate to reach out to us. We look forward to your feedback.

Thank you for your attention.

Sincerely,

Matheus Ramalho de Lima, professor

Formatted: English (United States)

Formatted: Justified

Below, we provide a point-by-point response to the issues raised:

~~**Comments to the Author**~~

~~5.~~ Review Comments to the Author

Please use the space provided to explain your answers to the questions above. You may also include additional comments for the author, including concerns about dual publication, research ethics, or publication ethics. (Please upload your review as an attachment if it exceeds 20,000 characters)

Reviewer #1: Manuscript PONE-D-23-09736, entitled “Replacement of Choline Chloride by a natural source Kolin Plus™ FC on the improvement of performance, economic viability, and reducing steatosis in broilers diets from 1 to 42 days of age”

Recommendation: The above paper is not suitable for publication in its present form.

General comments: adjust

1) The article provides useful information about the effects of replacement of choline chloride by a natural source Kolin Plus™ FC on performance, health parameters and economic viability in broilers. Although, the experiment was in general appropriately designed and implemented there are some points that should be corrected or clarified.
2) Please rephrase in Introduction L2-8, 19-21 and last paragraph.
3) Results should be presented as days 1-21, 22-42 and total 1-42
4) Tables 1-3 should be removed to supplementary materials
5) Some references are not included in reference list (Introduction Zeisel, 1991, Farina et al. 2017, L228: McDOWELL, 2000, L281: Bunchasak et al. (2016) and Crisol-Martínez et al. (2019), L287: Zhang et al. (2021). Some references are not cited in text: L319-321, 363-364, 420-423.

Specific comments:

Abstract
L1-2: This study aimed to evaluate the effects of natural choline (Kolin Plus™ FC) as a replacement for choline chloride on performance, blood variables and economic viability in broilers.” adjust
L3-4: “…of 6 replications of 25 one-day old male Ross chicks from…” Number of hybrid? adjust
L5: “was provided in” instead of “occurred under” adjust
L9: “assessed” instead of “measured” adjust
L13-14: “The results show a better result of supplementation with KPFC to CC and MAR in the broiler's diet from 1 to 42 days.” Please be specific. In which parameters? adjust
L16: What do you mean by “KPFC has an effective contribution to phosphatidylcholine”? adjust
L17-23: These findings are not yours. Please delete adjust
Where are the data referring to financial efficiency presented? adjust

Formatted: English (United States)
Formatted: English (United States)
Formatted: English (United States)
Formatted: English (United States)
Formatted: English (United States)
Formatted: English (United States)
Formatted: English (United States)
Formatted: English (United States)
Formatted: English (United States)

Introduction adjust
L1: “diets” instead of “feed”
L2: “contribute to” instead of “grow and in”
L18: “…its activity (Sherriff et al. 2016; Zhu et al. 2014).”
L21-22: Please delete “published in the Journal of Nutrition”
L23-24: Please delete “published in the American Journal of Clinical Nutrition”
L27: Please delete “A study published in the Journal of Nutrition found that”
L30: “The absence or deficiency of choline at under the recommended levels increases…”
L32: “provided” instead of “done”
L35: “allows us to observe”?
L46: “increases the reduction”?

Formatted: English (United States)

Housing conditions and management adjust
L4: “1050-day old male” or “1500 day-old male”?

Formatted: English (United States)

Animals adjust
L2: 39g or 45 g (as in abstract)?

Formatted: English (United States)

“Diets” instead of “Feed” adjust
L8: “are presented” instead of “were described”

Formatted: English (United States)

L35: “to estimate the delta”? adjust
L39: “…using the Folch method (1957), and lipids…”
L57: “in lower BW and less BWG” compared to?
L73: “…of Choline Chloride vs 300 g/t of the MAR product…”
L79: Are you sure? Same superscripts
L88: “…and FCR (improvement by 8 points; P = 0.0015) (Table 5).”
L89: “KPFC fed at 100 g/t improved FCR by 8 points” compared to?
L93: Please rephrase “effects did not differ significantly between treatments”
L161-166: Please remove this part to discussion
L226: ZEISEL or ZEIZEL?

Formatted: English (United States)

Reviewer #2: General: The objective of the experiment reported is to determine the impact of using three sources of choline chloride, each at three levels, and study effect in broilers using various responses. I must acknowledge the authors’ hard work in carrying out the experiment and following up with statistical analysis. Nevertheless, the manuscript has too many weaknesses, as pointed out below. The authors must first remember to put line numbers in their manuscripts. That will make the life of the reviewer much easier. The language used in the manuscript makes it difficult to follow the science. It distracts from the message and needs to be completely overhauled. The statistical analysis is inadequate and incorrectly done, considering how the treatments were arranged (see details below). The writing of the manuscript is rather untidy, making it look like it was haphazardly put together. The authors must clearly explain the choice of the levels of each choline chloride source they used.

Abstract adjust all points

Formatted: English (United States)

- The opening sentences do not read well.
- The sentence that begins with “KPFC has an…” and “Animals raised in conditions…” are totally out of place in the abstract or should be in an earlier part of the abstract.
- General comment. The abstract does not read well. I’m unsure about the statistical analysis, and the result presentation is not easy to follow. The abstract reads more like an opinion than a scientific abstract.

Introduction

Par 1 – the last sentence doesn’t read well. it needs to be rewritten
Par 2 – some of the references are not formatted correctly
Par 3 - reads like what you would see in a newspaper, not a scientific article.
L22: what modifications?
L24: indicate the temperature in which the samples were kept
L25: historical? What is that?
L26-28: this quantification method is totally subjective
L49:50: this provides pairwise comparison. I think several things are wrong here. First, the data should be analyzed as a 3x3 plus 1 factorial (factors are source and level of choline chloride, plus a control), which agrees with the treatment arrangement. with that in mind, the data can be analyzed with the control nested within the factors. If statistical analysis reveals interactions, the levels within each source of choline can be separated (means separation) using orthogonal polynomial contrast (linear or quadratic contrasts). The pairwise comparisons done here is akin to multiple linear comparison, like Duncan’s which is both inappropriate and weak.
L54-58: the presentation of this data is almost impossible to follow. These p values are not relevant and not appropriate for the design. The conclusion can not be relied on.
Results section: this section needs to be rewritten after the data are analyzed correctly.
Discussion section: please see previous comment. The discussion should address advancement in knowledge that this research presents. It should follow an appropriate stat analysis. It is not doing that in the current state.
Table 4-8:
- what does the column “live” in some of the tables mean?
- The data need to be reanalyzed. The multitudinous orthogonal contrasts are meaningless and do not help the reader at all
Tables 9 and 10 – I don’t think these tables and data are of value in this kind of scientific writing. There are appropriate journals where economic data can be presented. Please delete them and writing based on them.

Reviewer #3: The research article “PONE-D-23-09736: Replacement of Choline Chloride by a natural source Kolin Plus™ FC on the improvement of performance, economic viability, and reducing steatosis in broilers diets from 1 to 42 days of age” compares different Choline Chloride sources in boilers. The topic is interesting and can add value to the existing knowledge in this area. However, the way the data is analysed and the results presented do not confirm the conclusion drawn. Too many details are missing in the manuscripts. There are fundamental issues with the article's statistical soundness. It is therefore strongly advised to consult with a statistical expert to improve further the manuscript integrity and reliability of your data analysis.

Some of the major points that need to be revisited and revised in the manuscript are as below:
Title: The title does not reflect the research conducted. The research compares different sources of Choline chloride, whereas the tile only highlights one source. Consider revision.

Abstract: “This study aimed to evaluate natural choline (Kolin Plus™ FC) as a replacement for choline chloride”. This statement is very confusing. In this study, a comparison is made between the three sources of Choline chloride. This is a comparative study. Were the other two sources already evaluated? If yes, give a reference. adjust

“The Control treatment was not supplemented with choline source”. The statement is not clear. All experimental diets, including the basal diet, show the presence of choline (calculated Choline content, see tables 1, 2 and 3). If the basal group had no supplemental choline chloride there, where this calculated value came from? Clarify.

“Animals raised in conditions of greater predisposition to heat stress can suffer oxidative stress, KPFC has compounds that, in addition to reducing free radicals, inhibit TRPM2 channels in hepatocytes, thus allowing, in reduction of lipid deposition, reduced gene expression of cytokines (IL-1β and IL-6), which generates better cell protection”. Not sure why the authors are referring to heat stress and oxidative stress. There was no heat stress in this study. Revise the sentence to clarify.

“In conclusion, KPFC supplementation is more efficient than CC and MAR with improved zootechnical performance and reduces hepatic steatosis in broilers from 1 to 42 days” . Unfortunately, this statement is not true. The effect of KPFC did not show improvement at all phases and in all parameters, so revise the sentence to reflect where significant improvements were noted. adjust

Formatted: English (United States)

Formatted: English (United States)

Introduction Section:
“potential substitutes for synthetic source”: Mention names of synthetic sources.
“TRPM2”: write full word when first used, followed by the abbreviation in the brackets.
“This study was carried out to compare the diet of broilers replacing choline supplementation by a synthetic source to a natural sources considering the effects on performance, carcass quality, blood parameters, and steatosis in the liver of broilers from 1 to 42 days of age”. The study object is not clear. Revise the sentence and highlight the study hypothesis. adjust

Formatted: English (United States)

Material and Methods section:
“UFPB”: write full word when first used, followed by the abbreviation in the brackets.
Mention temp and humidity range during the trial period. Also, mention the duration of light and darkness. adjust
What was the form of the feed? Mash, crumbs or pelleted. adjust
Is basal diet treated as control group? If yes, where choline chroide came from in that diet (see Table 1, 2, 3). adjust
How was choline chloride added in the diet? Was it part of the premix? Were all sources of choline chloride in the form of powder? adjust

Formatted: English (United States)

Formatted: English (United States)

Formatted: English (United States)

Formatted: English (United States)

“Diets were representative of local commercial feed formulations and designed to meet or exceeded nutritional requirements for broiler strain and age”. Do you mean Cobb Nutrition guidelines were used? If yes then mention it for clarity.
Table 1: The basal and all three CC diets have similar calculated Choline levels, i.e. 1,162 mg/kg. If yes, then why should we expect a difference in performance? Not clear?
Performance: Line 7 to 11 is a repeat of information already provided in the dietary treatments section. Delete these lines from this section.
Carcass: Add formulas used to calculate carcass composition.
Fatty liver data: Line 19: which tissue?
Line 23: Why and how much talc was added? add reference
Economic analysis: mention the formula for how the economic analysis was calculated. Not clear.
Statistical analysis:, adjust all points

Formatted: English (United States)

Inadequate Sample Size and Power: The sample size (6 replicate pens only) used in the study appears to be insufficient for the research questions addressed. As a result, the statistical power of the analysis may be compromised, leading to unreliable results. The authors should address this limitation by justifying the chosen sample size.
The article lacks the application of appropriate statistical tests to evaluate the significance of the reported findings. Claims and conclusions are drawn without statistical evidence, raising doubts about the validity of the results. I recommend that the authors reanalyse the data using relevant statistical tests to support their assertions adequately.
The authors are strongly advised to consult with a statistical expert to ensure that a robust test model is used to determine the best source of Choline chlorine and an optimum dosage for each phase and parameter.
Results tables are very difficult to interpret as orthogonal contrast only allows specific comparisons, especially when the treatment P-Value is non-significant.
Table 5 should represent the grower phase, not day1 to 35.
How birds performed in the finisher phase is also missing? Add a table
Why were two overall periods presented from day 1 to 35 and d1 to 42? Not clear what the purpose was?
The way data is analysed makes it difficult to assess the overall effect of sources and doses. Maybe consider factorial design? 3 sources x 3 levels(low, medium and high) +1control.

Discussion:, adjust

Formatted: English (United States)

“In field conditions with challenges such as low quality of ingredients, heat stress, and high density of animals, higher doses of the KPFC evaluated in this study (200g/t or 300g/t) can be recommended”. How can you recommend something which is not tested in the current study?? . Revise sentence
“From a practical standpoint (i.e., product 304 storage and feed logistics) the lower dose requirement is another advantage of KPFC relative to Choline Chloride, the commercial doses recommended of CC (400, 800, or 1,200 g/t) can involve several space-related and logistics concerns.” Again which space-related and logistic concerns? This is not something tested or discussed in this study. Revise sentence

Conclusion: The conclusion section needs to be rewritten after appropriate statistical analysis is conducted. The current conclusions do not reflect the current objectives.
Natural Choline KPFC is a safe source of choline: What do you mean by safe? Not clear. Did other sources cause mortality or toxicity? adjust
"Natural Choline KPFC is recommended in low oxidative stress 314 conditions. In higher exposure to heat stress, it is recommended doses of 200g/ton or 300g/t 315 of Natural Choline KPFC in diet to broilers from 1 to 42 days of age". This study had no oxidative stress model, so how can you recommend doses for low and high oxidative stress?
There are fundamental issues with the article's statistical soundness. It is strongly recommended that the authors conduct a considerable revision addressing the abovementioned points. adjust

Formatted: English (United States)

Formatted: English (United States)

Reviewer #4: Dear Author adjust
As a result of the examination I have made, I am of the opinion that the work in its current form is not in accordance with the journal writing policy.

Formatted: English (United States)

---

6. PLOS authors have the option to publish the peer review history of their article (what does this mean?). If published, this will include your full peer review and any attached files.

Field Code Changed

If you choose "no", your identity will remain anonymous but your review may still be made public.

Do you want your identity to be public for this peer review? For information about this choice, including consent withdrawal, please see our Privacy Policy.

Field Code Changed

Reviewer #1: No

Reviewer #2: No

Reviewer #3: No

Reviewer #4: No
