# Supplementary material for: Choline supplementation: Impact on broiler chicken performance, steatosis, and economic viability from from 1 to 42 days
Source: PLoS One. 2024 Mar 19;19(3):e0295488. doi: 10.1371/journal.pone.0295488 (PMC10950236; doi:10.1371/journal.pone.0295488)
Supplement: Attachment — Submitted filename: Comments to the Author_.docx [file pone.0295488.s002.docx]

Dear Reviewers,

Thank you for your thorough evaluation of our manuscript titled “Choline through Diet or Supplementation? Synthetic or Natural Source Supplementation? Improving Performance, Reducing Steatosis, and Economic Viability of Broiler Chickens from 1 to 42 Days.” We appreciate your valuable feedback and have made the necessary revisions based on your comments.

We apologize for any oversight regarding the similarity to the previously submitted manuscript (PONE-D-23-09736). We acknowledge that this is indeed a resubmission.

We hope that these changes address the concerns raised during the initial review. Please find the revised manuscript attached. We appreciate your time and effort in evaluating our work.

Significant adjustments have been made to the document, and the addressed items were met punctually. Additionally, points that were not previously included in the discussion have been added. Therefore, some aspects do not have a direct response to the specific item, as the context of our revision has evolved. We conducted a broader edit, considering the points raised, and in some cases, even altering the structure from the previously submitted version.

If you have any further suggestions or require additional clarifications, please do not hesitate to reach out to us. We look forward to your feedback.

Thank you for your attention.

Sincerely,

Matheus Ramalho de Lima, professor

Below, we provide a point-by-point response to the issues raised:

Review Comments to the Author

Please use the space provided to explain your answers to the questions above. You may also include additional comments for the author, including concerns about dual publication, research ethics, or publication ethics. (Please upload your review as an attachment if it exceeds 20,000 characters)

Reviewer #1: Manuscript PONE-D-23-09736, entitled “Replacement of Choline Chloride by a natural source Kolin Plus™ FC on the improvement of performance, economic viability, and reducing steatosis in broilers diets from 1 to 42 days of age”

Recommendation: The above paper is not suitable for publication in its present form.


General comments: adjust

1) The article provides useful information about the effects of replacement of choline chloride by a natural source Kolin Plus™ FC on performance, health parameters and economic viability in broilers. Although, the experiment was in general appropriately designed and implemented there are some points that should be corrected or clarified.
2) Please rephrase in Introduction L2-8, 19-21 and last paragraph.
3) Results should be presented as days 1-21, 22-42 and total 1-42
4) Tables 1-3 should be removed to supplementary materials
5) Some references are not included in reference list (Introduction Zeisel, 1991, Farina et al. 2017, L228: McDOWELL, 2000, L281: Bunchasak et al. (2016) and Crisol-Martínez et al. (2019), L287: Zhang et al. (2021). Some references are not cited in text: L319-321, 363-364, 420-423.

Specific comments:


Abstract
L1-2: This study aimed to evaluate the effects of natural choline (Kolin Plus™ FC) as a replacement for choline chloride on performance, blood variables and economic viability in broilers.” adjust
L3-4: “…of 6 replications of 25 one-day old male Ross chicks from…” Number of hybrid? adjust
L5: “was provided in” instead of “occurred under” adjust
L9: “assessed” instead of “measured” adjust
L13-14: “The results show a better result of supplementation with KPFC to CC and MAR in the broiler's diet from 1 to 42 days.” Please be specific. In which parameters? adjust
L16: What do you mean by “KPFC has an effective contribution to phosphatidylcholine”? adjust
L17-23: These findings are not yours. Please delete adjust
Where are the data referring to financial efficiency presented? adjust

Introduction adjust
L1: “diets” instead of “feed”
L2: “contribute to” instead of “grow and in”
L18: “…its activity (Sherriff et al. 2016; Zhu et al. 2014).”
L21-22: Please delete “published in the Journal of Nutrition”
L23-24: Please delete “published in the American Journal of Clinical Nutrition”
L27: Please delete “A study published in the Journal of Nutrition found that”
L30: “The absence or deficiency of choline at under the recommended levels increases…”
L32: “provided” instead of “done”
L35: “allows us to observe”?
L46: “increases the reduction”?

Housing conditions and management adjust
L4: “1050-day old male” or “1500 day-old male”?

Animals adjust
L2: 39g or 45 g (as in abstract)?

“Diets” instead of “Feed” adjust
L8: “are presented” instead of “were described”

L35: “to estimate the delta”? adjust
L39: “…using the Folch method (1957), and lipids…”
L57: “in lower BW and less BWG” compared to?
L73: “…of Choline Chloride vs 300 g/t of the MAR product…”
L79: Are you sure? Same superscripts
L88: “…and FCR (improvement by 8 points; P = 0.0015) (Table 5).”
L89: “KPFC fed at 100 g/t improved FCR by 8 points” compared to?
L93: Please rephrase “effects did not differ significantly between treatments”
L161-166: Please remove this part to discussion
L226: ZEISEL or ZEIZEL?

Reviewer #2: General: The objective of the experiment reported is to determine the impact of using three sources of choline chloride, each at three levels, and study effect in broilers using various responses. I must acknowledge the authors’ hard work in carrying out the experiment and following up with statistical analysis. Nevertheless, the manuscript has too many weaknesses, as pointed out below. The authors must first remember to put line numbers in their manuscripts. That will make the life of the reviewer much easier. The language used in the manuscript makes it difficult to follow the science. It distracts from the message and needs to be completely overhauled. The statistical analysis is inadequate and incorrectly done, considering how the treatments were arranged (see details below). The writing of the manuscript is rather untidy, making it look like it was haphazardly put together. The authors must clearly explain the choice of the levels of each choline chloride source they used.

Abstract adjust all points
- The opening sentences do not read well.
- The sentence that begins with “KPFC has an…” and “Animals raised in conditions…” are totally out of place in the abstract or should be in an earlier part of the abstract.
- General comment. The abstract does not read well. I’m unsure about the statistical analysis, and the result presentation is not easy to follow. The abstract reads more like an opinion than a scientific abstract.
Introduction
Par 1 – the last sentence doesn’t read well. it needs to be rewritten
Par 2 – some of the references are not formatted correctly
Par 3 - reads like what you would see in a newspaper, not a scientific article.
L22: what modifications?
L24: indicate the temperature in which the samples were kept
L25: historical? What is that?
L26-28: this quantification method is totally subjective
L49:50: this provides pairwise comparison. I think several things are wrong here. First, the data should be analyzed as a 3x3 plus 1 factorial (factors are source and level of choline chloride, plus a control), which agrees with the treatment arrangement. with that in mind, the data can be analyzed with the control nested within the factors. If statistical analysis reveals interactions, the levels within each source of choline can be separated (means separation) using orthogonal polynomial contrast (linear or quadratic contrasts). The pairwise comparisons done here is akin to multiple linear comparison, like Duncan’s which is both inappropriate and weak.
L54-58: the presentation of this data is almost impossible to follow. These p values are not relevant and not appropriate for the design. The conclusion can not be relied on.
Results section: this section needs to be rewritten after the data are analyzed correctly.
Discussion section: please see previous comment. The discussion should address advancement in knowledge that this research presents. It should follow an appropriate stat analysis. It is not doing that in the current state.
Table 4-8:
- what does the column “live” in some of the tables mean?
- The data need to be reanalyzed. The multitudinous orthogonal contrasts are meaningless and do not help the reader at all
Tables 9 and 10 – I don’t think these tables and data are of value in this kind of scientific writing. There are appropriate journals where economic data can be presented. Please delete them and writing based on them.

Reviewer #3: The research article “PONE-D-23-09736: Replacement of Choline Chloride by a natural source Kolin Plus™ FC on the improvement of performance, economic viability, and reducing steatosis in broilers diets from 1 to 42 days of age” compares different Choline Chloride sources in boilers. The topic is interesting and can add value to the existing knowledge in this area. However, the way the data is analysed and the results presented do not confirm the conclusion drawn. Too many details are missing in the manuscripts. There are fundamental issues with the article's statistical soundness. It is therefore strongly advised to consult with a statistical expert to improve further the manuscript integrity and reliability of your data analysis.

Some of the major points that need to be revisited and revised in the manuscript are as below:
Title: The title does not reflect the research conducted. The research compares different sources of Choline chloride, whereas the tile only highlights one source. Consider revision.

Abstract: “This study aimed to evaluate natural choline (Kolin Plus™ FC) as a replacement for choline chloride”. This statement is very confusing. In this study, a comparison is made between the three sources of Choline chloride. This is a comparative study. Were the other two sources already evaluated? If yes, give a reference. adjust
“The Control treatment was not supplemented with choline source”. The statement is not clear. All experimental diets, including the basal diet, show the presence of choline (calculated Choline content, see tables 1, 2 and 3). If the basal group had no supplemental choline chloride there, where this calculated value came from? Clarify.
“Animals raised in conditions of greater predisposition to heat stress can suffer oxidative stress, KPFC has compounds that, in addition to reducing free radicals, inhibit TRPM2 channels in hepatocytes, thus allowing, in reduction of lipid deposition, reduced gene expression of cytokines (IL-1β and IL-6), which generates better cell protection”. Not sure why the authors are referring to heat stress and oxidative stress. There was no heat stress in this study. Revise the sentence to clarify.
“In conclusion, KPFC supplementation is more efficient than CC and MAR with improved zootechnical performance and reduces hepatic steatosis in broilers from 1 to 42 days” . Unfortunately, this statement is not true. The effect of KPFC did not show improvement at all phases and in all parameters, so revise the sentence to reflect where significant improvements were noted. adjust

Introduction Section:
“potential substitutes for synthetic source”: Mention names of synthetic sources.
“TRPM2”: write full word when first used, followed by the abbreviation in the brackets.
“This study was carried out to compare the diet of broilers replacing choline supplementation by a synthetic source to a natural sources considering the effects on performance, carcass quality, blood parameters, and steatosis in the liver of broilers from 1 to 42 days of age”. The study object is not clear. Revise the sentence and highlight the study hypothesis. adjust

Material and Methods section:
“UFPB”: write full word when first used, followed by the abbreviation in the brackets.
Mention temp and humidity range during the trial period. Also, mention the duration of light and darkness. adjust
What was the form of the feed? Mash, crumbs or pelleted. adjust
Is basal diet treated as control group? If yes, where choline chroide came from in that diet (see Table 1, 2, 3). adjust
How was choline chloride added in the diet? Was it part of the premix? Were all sources of choline chloride in the form of powder? adjust
“Diets were representative of local commercial feed formulations and designed to meet or exceeded nutritional requirements for broiler strain and age”. Do you mean Cobb Nutrition guidelines were used? If yes then mention it for clarity.
Table 1: The basal and all three CC diets have similar calculated Choline levels, i.e. 1,162 mg/kg. If yes, then why should we expect a difference in performance? Not clear?
Performance: Line 7 to 11 is a repeat of information already provided in the dietary treatments section. Delete these lines from this section.
Carcass: Add formulas used to calculate carcass composition.
Fatty liver data: Line 19: which tissue?
Line 23: Why and how much talc was added? add reference
Economic analysis: mention the formula for how the economic analysis was calculated. Not clear.
Statistical analysis: adjust all points
Inadequate Sample Size and Power: The sample size (6 replicate pens only) used in the study appears to be insufficient for the research questions addressed. As a result, the statistical power of the analysis may be compromised, leading to unreliable results. The authors should address this limitation by justifying the chosen sample size.
The article lacks the application of appropriate statistical tests to evaluate the significance of the reported findings. Claims and conclusions are drawn without statistical evidence, raising doubts about the validity of the results. I recommend that the authors reanalyse the data using relevant statistical tests to support their assertions adequately.
The authors are strongly advised to consult with a statistical expert to ensure that a robust test model is used to determine the best source of Choline chlorine and an optimum dosage for each phase and parameter.
Results tables are very difficult to interpret as orthogonal contrast only allows specific comparisons, especially when the treatment P-Value is non-significant.
Table 5 should represent the grower phase, not day1 to 35.
How birds performed in the finisher phase is also missing? Add a table
Why were two overall periods presented from day 1 to 35 and d1 to 42? Not clear what the purpose was?
The way data is analysed makes it difficult to assess the overall effect of sources and doses. Maybe consider factorial design? 3 sources x 3 levels(low, medium and high) +1control.

Discussion: adjust
“In field conditions with challenges such as low quality of ingredients, heat stress, and high density of animals, higher doses of the KPFC evaluated in this study (200g/t or 300g/t) can be recommended”. How can you recommend something which is not tested in the current study?? . Revise sentence
“From a practical standpoint (i.e., product 304 storage and feed logistics) the lower dose requirement is another advantage of KPFC relative to Choline Chloride, the commercial doses recommended of CC (400, 800, or 1,200 g/t) can involve several space-related and logistics concerns.” Again which space-related and logistic concerns? This is not something tested or discussed in this study. Revise sentence

Conclusion: The conclusion section needs to be rewritten after appropriate statistical analysis is conducted. The current conclusions do not reflect the current objectives.
Natural Choline KPFC is a safe source of choline: What do you mean by safe? Not clear. Did other sources cause mortality or toxicity? adjust
“Natural Choline KPFC is recommended in low oxidative stress 314 conditions. In higher exposure to heat stress, it is recommended doses of 200g/ton or 300g/t 315 of Natural Choline KPFC in diet to broilers from 1 to 42 days of age”. This study had no oxidative stress model, so how can you recommend doses for low and high oxidative stress?
There are fundamental issues with the article's statistical soundness. It is strongly recommended that the authors conduct a considerable revision addressing the abovementioned points. adjust

Reviewer #4: Dear Author adjust
As a result of the examination I have made, I am of the opinion that the work in its current form is not in accordance with the journal writing policy.
